# Whole-exome sequencing in patients with protein aggregate myopathies reveals causative mutations associated with novel atypical phenotypes

**DOI:** 10.1007/s10072-020-04876-7

**Published:** 2020-11-10

**Authors:** Marcin M. Machnicki, Valeria Guglielmi, Elia Pancheri, Francesca Gualandi, Lorenzo Verriello, Katarzyna Pruszczyk, Joanna Kosinska, Antonella Sangalli, Malgorzata Rydzanicz, Maria Grazia Romanelli, Marcella Neri, Rafal Ploski, Paola Tonin, Giuliano Tomelleri, Tomasz Stoklosa, Gaetano Vattemi

**Affiliations:** 1 Department of Immunology, Medical University of Warsaw, Warsaw, Poland; 2 Department of Neurosciences, Biomedicine and Movement Sciences, Section of Clinical Neurology, University of Verona, Verona, Italy; 3 UOL of Medical Genetics, Department of Medical Science, University of Ferrara, Ferrara, Italy; 4 Neurologic Clinic, Department of Neurological Sciences, Azienda Sanitaria Universitaria Integrata di Udine, Udine, Italy; 5 Department of Medical Genetics, Medical University of Warsaw, Warsaw, Poland; 6 Department of Neurosciences, Biomedicine and Movement Sciences, Section of Biology and Genetics, University of Verona, Verona, Italy

**Keywords:** Protein aggregate myopathies, Myofibrillar myopathies, Whole-exome sequencing, *LMNA*, *RYR1*, *TTN*

## Abstract

**Background:**

Myofibrillar myopathies (MFM) are a subgroup of protein aggregate myopathies (PAM) characterized by a common histological picture of myofibrillar dissolution, Z-disk disintegration, and accumulation of degradation products into inclusions. Mutations in genes encoding components of the Z-disk or Z-disk-associated proteins occur in some patients whereas in most of the cases, the causative gene defect is still unknown. We aimed to search for pathogenic mutations in genes not previously associated with MFM phenotype.

**Methods:**

We performed whole-exome sequencing in four patients from three unrelated families who were diagnosed with PAM without aberrations in causative genes for MFM.

**Results:**

In the first patient and her affected daughter, we identified a heterozygous p.(Arg89Cys) missense mutation in *LMNA* gene which has not been linked with PAM pathology before. In the second patient, a heterozygous p.(Asn4807Phe) mutation in *RYR1* not previously described in PAM represents a novel, candidate gene with a possible causative role in the disease. Finally, in the third patient and his symptomatic daughter, we found a previously reported heterozygous p.(Cys30071Arg) mutation in *TTN* gene that was clinically associated with cardiac involvement.

**Conclusions:**

Our study identifies a new genetic background in PAM pathology and expands the clinical phenotype of known pathogenic mutations.

**Supplementary Information:**

The online version contains supplementary material available at 10.1007/s10072-020-04876-7.

## Introduction

Myofibrillar myopathies (MFM) are a group of protein aggregate myopathies (PAM) sharing the histological features of Z-disk dissolution, myofibrillar degeneration, and accumulation of degradation products into protein aggregates [1–4]. The clinical spectrum is wide and consists mainly in progressive muscle weakness of upper and/or lower limbs; limb-girdle and scapuloperoneal phenotypes can be observed as well as involvement of hand, facial, pharyngeal, and respiratory muscles [1–4]. Cardiomyopathy, peripheral neuropathy, and cataract are frequent associated conditions [1–4]. The diagnosis is established by muscle biopsy which shows as main morphological hallmark, abnormal fibers containing amorphous material of irregular shape and size positive to several proteins including αB-crystallin, desmin, and myotilin [1–3]. MFM are usually transmitted as an autosomal dominant trait; however, X-linked, autosomal recessive and sporadic cases have been described [1–4]. Causative mutations have been identified in a minority of patients in one of the following genes: desmin (*DES*), αB-crystallin (*CRYAB*), myotilin (*MYOT*), Z-band alternatively spliced PDZ-containing protein (*LBD3/ZASP*), Bcl2-associated athanogene-3 (*BAG3*), and filamin C (*FLNC*) [1–5]. Recent mutations in *FHL1*, *TTN*, *DNAJB6*, *PLEC*, *ACTA1*, *HSPB8*, *LMNA*, *KY*, *PYROXD1*, and *SQSTM1* have also been reported in patients featuring MFM pathology highlighting the variability and complexity of these muscular disorders [5].

We performed whole-exome sequencing (WES) in four patients with PAM from three unrelated families in whom classical genetic approach had failed to identify a causative mutation in one of the known MFM causing genes.

## Materials and methods

### Patients

**Fig. 1:**
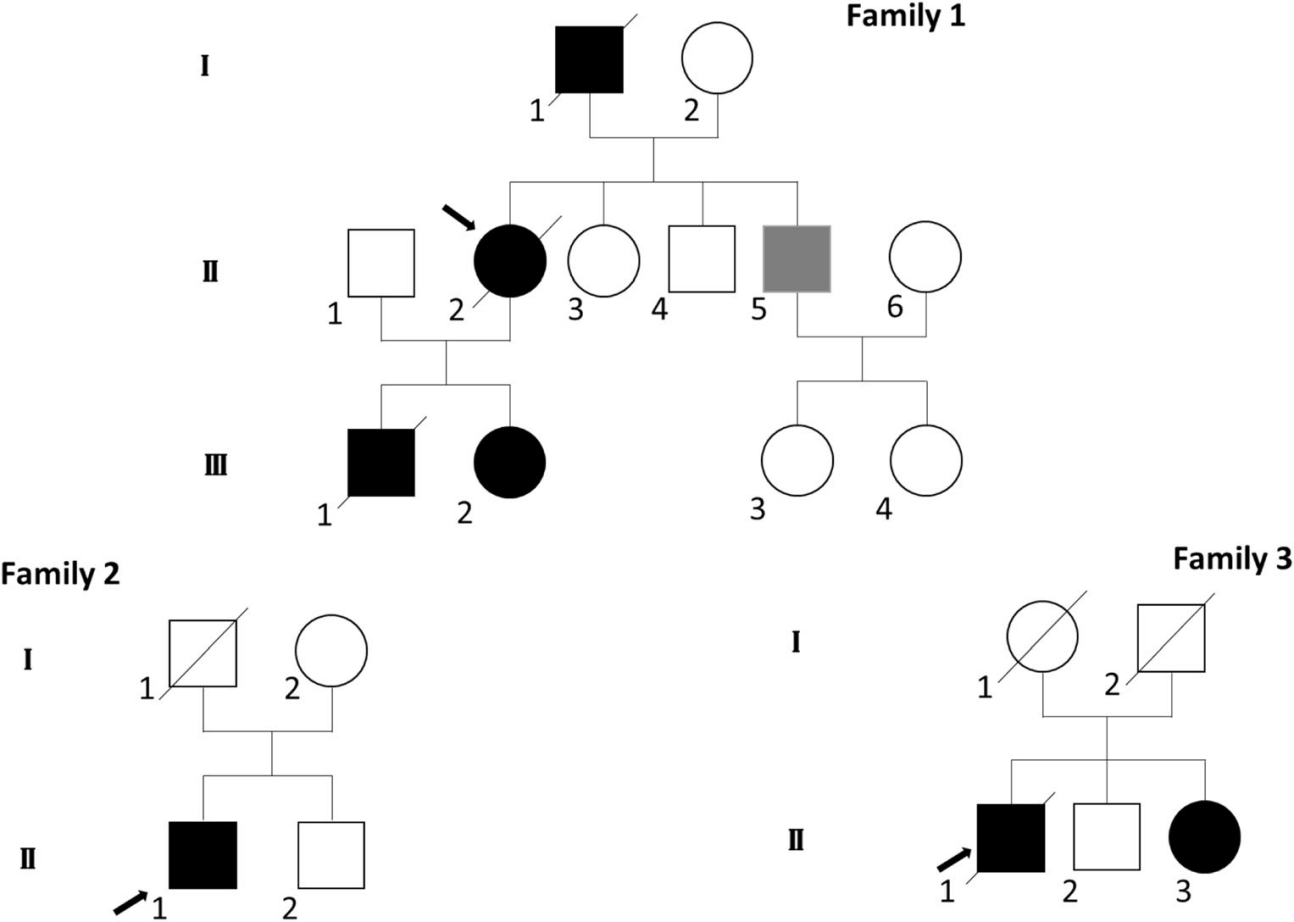
Pedigrees of the three families. Arrows indicate the proband in each pedigree. Solid black symbols denote affected family members. Gray solid symbol represents patient affected by myopathy but not carrying p.(Arg89Cys) *LMNA* mutation

We studied three unrelated families in which the probands diagnosed with PAM had negative results by gene testing for MFM including *DES*, *CRYAB*, *MYOT*, *LBD3/ZASP*, *BAG3*, and *FLNC* (Fig. 1). The diagnosis of MFM was established according to the clinical and morphological criteria provided by Schröder and Schoser [2]. Muscle biopsies were performed for diagnostic purpose. All patients provided written informed consent to the study, which was approved by our local Ethics Committee and conducted in accordance with the ethical guidelines of the Declaration of Helsinki.

### Immunohistochemical studies

Immunohistochemistry was performed on 8-μm-thick muscle sections with antibodies to desmin, αB-crystallin, myotilin, and lamin A/C. The reactions were revealed by immunofluorescence methods as previously described [4]. Hoechst 33258 staining was used to depict cell nuclei.

### Genetic investigations

Whole-exome sequencing (WES) was performed on four affected members of three families (Family 1:II-2 and III-2, Family 2:II-1, and Family 3:II-2). Genomic DNA libraries were prepared using the KAPA HTP library preparation kit, multiplexed to 8-plex pools prior to the SeqCap EZ Exome v2.0 capture (Roche NimbleGen) and sequenced on the Illumina HiSeq platform (2 × 100-bp reads). Mean coverage in range 26.07–34.39x and ge10 in range 48.9–51.1% were achieved. One sample was captured using MedExome probes (Roche NimbleGen) and sequenced to reach 93.87x mean coverage and 96.9% ge20. All steps were carried out according to original protocols.

Variant discovery included the following steps: quality control of raw FASTQ, adapter trimming, and low-quality reads removal using Trimmomatic, read mapping to hg19 genome using BWA, duplication removal, local realignment, and quality recalibration using GATK and Picard, and variant calling using UnifiedGenotyper, HaplotypeCaller, and FreeBayes [6–9].

Variants were filtered using public (NHLBI ESP, gnomAD) and internal databases in order to remove common genetic variation [10, 11]. CADD, PolyPhen2, SIFT, FATHMM, and MutationTaster were used to identify possible protein-damaging variants [12–16]. Variants were classified using ClinVar and VarSome databases [17, 18].

Selected variants were detected independently using the Sanger sequencing using BigDye Chemistry (Applied Biosystems, Foster City, CA) on Genetic Analyzer 3500 (Applied Biosystems).

## Results

### Clinical, laboratory, and pathology data

#### Family 1

**Fig. 2:**
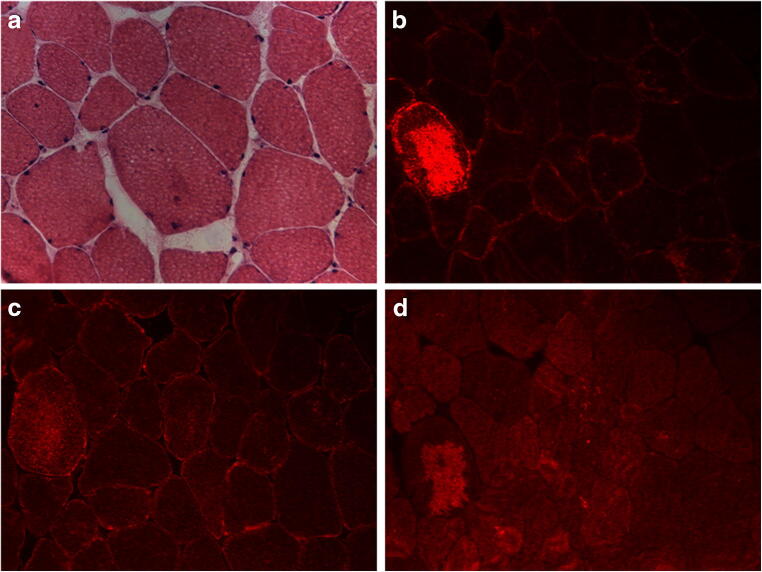
Muscle biopsy findings from proband of Family 1. H&E stained section (**a**) shows a muscle fiber containing a small focus of amorphous material, which strongly immunoreacts for αB-crystallin (**b**), desmin (**c**), and myotilin (**d**). Images were obtained with obj × 20

A 64-year-old Caucasian woman (II-2) reported a 6-month history of progressive muscle weakness with difficulty in climbing stairs and lifting weights. At age 33 years, an arrhythmogenic cardiomyopathy was diagnosed, and 14 years later, heart transplantation was performed. Past medical history was remarkable for type 2 diabetes mellitus, hypothyroidism, chronic kidney disease, and right radical nephrectomy for renal oncocytoma. At the time of clinical evaluation, she was able to walk for short distances and had moderate to marked weakness of proximal four-limb muscles with a mild involvement of distal muscles of the upper limbs. Deep tendon reflexes, sensation, and cerebellar examination were normal. The patient did not have respiratory symptoms and facial muscles were spared. Serum creatine kinase (sCK) was within the reference range while electromyography (EMG) showed diffuse myopathic changes. An open biopsy of left deltoid muscle showed increased fiber size variation, rare rimmed vacuoles, slight endomysial fibrosis, rare fibers containing amorphous material, and several fibers with patchy areas of reduced or increased staining of oxidative enzymes (Fig. S1). Abnormal muscle fibers had an ectopic accumulation of αB-crystallin, desmin, and myotilin by immunohistochemistry (Fig. 2). The patient died at age 67 years from unrelated causes.

Her father (I-1) and paternal grandmother had similar heart disease. Her son (III-1) had died at the age of 20 due to cardiomyopathy. Her 41-year-old daughter (III-2) presented from the age of 18 years an arrhythmogenic cardiomyopathy for which she first necessitated pacemaker implantation and then radiofrequency ablation for atrial flutter. From the age of 36, she developed mild proximal lower limbs muscle weakness with waddling gait. The proband had one healthy sister (II-3) and two brothers, one of which, aged 73 years (II-5), presented severe weakness of proximal limb muscles started at the age of 69 years. He also suffered from atrial fibrillation, diabetes mellitus, and arterial hypertension, which give a clinically different picture as compared to the affected patient and her daughter.

#### Family 2

**Fig. 3:**
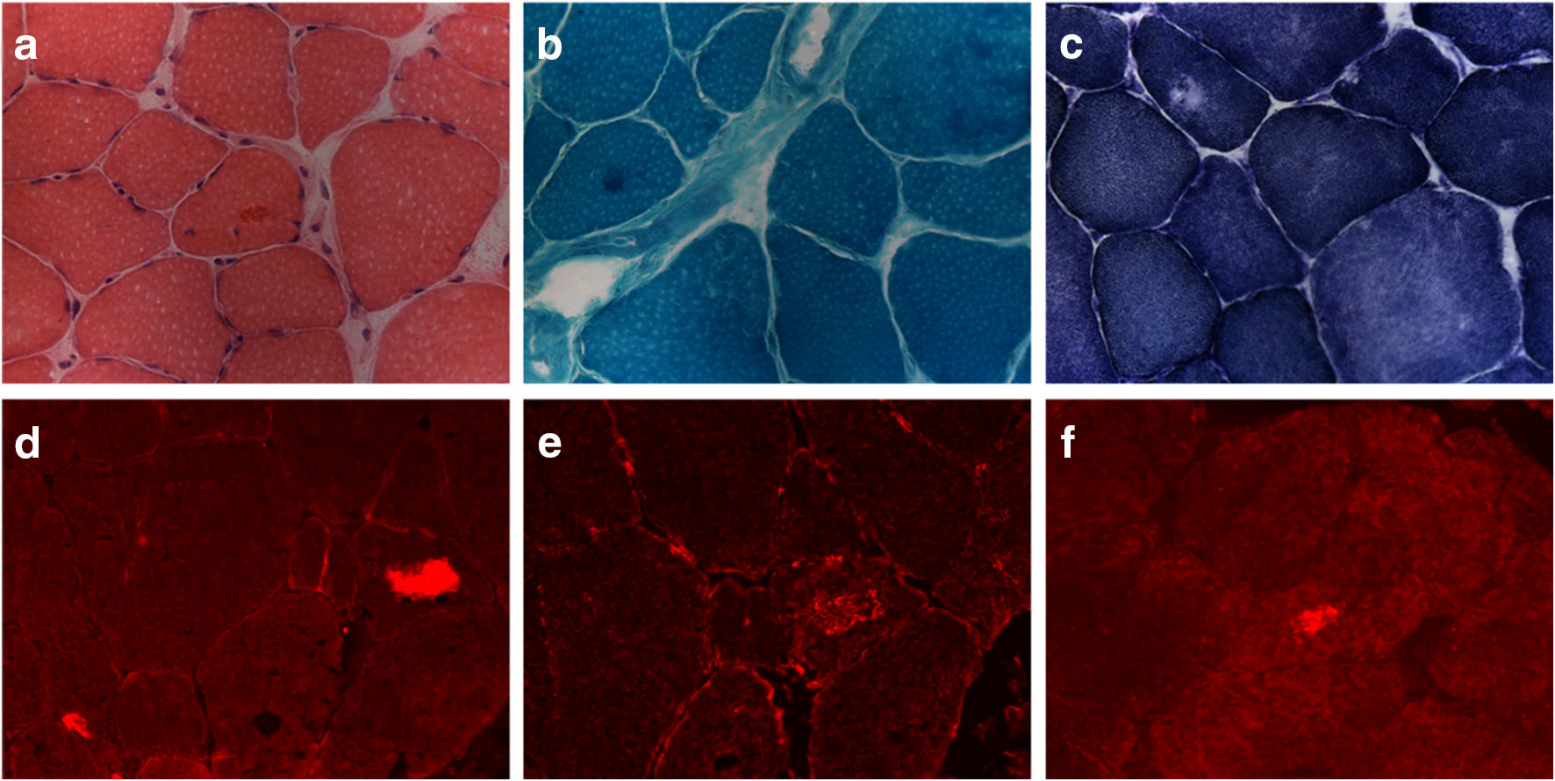
Muscle biopsy findings from proband of Family 2. H&E (**a**) and GT (**b**) show a muscle fiber harboring amorphous material. NADH staining (**c**) shows a fiber with focal decrease activity. Muscle fiber with strong immunoreactivity for αB-crystallin (**d**), desmin (**e**), and myotilin (**f**). Images were obtained with obj × 20

A 42-year-old Caucasian man (II-1) was referred for diffuse myalgia both at rest and exercise-related and gait disturbances started 1 month earlier. Past medical history was notable for atrial fibrillation requiring ablation. On neurological examination, he presented difficulty in walking on toes and heels with mild weakness and wasting of tibialis anterior muscles and bilateral pes cavus. Strength was normal in the remaining muscle groups. Ankle jerks were absent, while sensory, cerebellar, and cranial nerve examination were normal. sCK level was in the normal range and EMG recorded a mixed pattern with both neuropathic and myopathic changes in distal lower limb muscles. The patient underwent a biopsy of left tibialis anterior muscle that showed increased variation of fiber size with atrophic and hypertrophic fibers, fiber splitting, rare sarcoplasmic masses and ring fibers, slight increase of endomysial connective tissue, and fibers containing amorphous material that was eosinophilic by hematoxylin and eosin (H&E) and dark blue by modified Gomori trichrome (GT) (Fig. 3 and S2). A single fiber harbored cytoplasmic bodies. Immunohistochemical studies demonstrated the focal accumulation of desmin, αB-crystallin, and myotilin within abnormal muscle fibers (Fig. 3). Family history was unremarkable. The patient had one brother who was asymptomatic (II-2).

#### Family 3

**Fig. 4:**
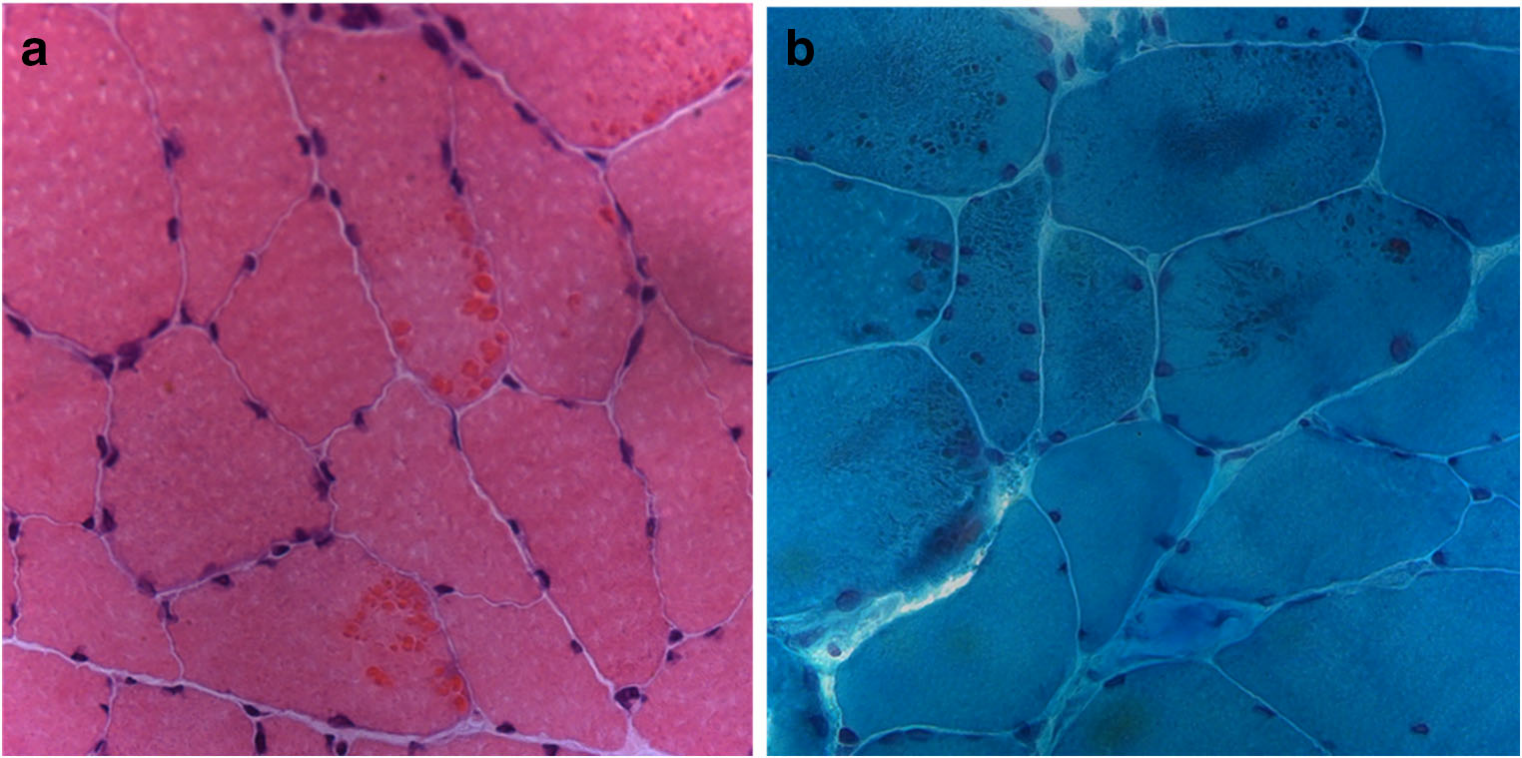
Muscle biopsy findings from proband of Family 3. Muscle fibers with amorphous material that stains eosinophilic on H&E (**a**) and dark blue on GT (**b**). Images were obtained with obj × 20

A 45-year-old Caucasian man (II-1) reported a 1-year history of progressive muscle weakness of the lower limbs with difficulty in climbing stairs. Shortly, he developed severe dyspnea requiring hospitalization in the intensive care unit for acute hypercapnic respiratory failure that necessitated first endotracheal intubation and invasive mechanical ventilation then nocturnal non-invasive ventilation. His past medical history was significant for type 2 diabetes mellitus and blood hypertension. On physical examination, the patient had a waddling and steppage gait and could not lift his arms above the head. There was moderate symmetric muscle weakness of shoulder abductors, hip flexors, hip abductors, and foot extensors, associated to mild proximal muscle wasting. Sensory, cerebellar, and cranial nerve examination and deep tendon reflexes were normal. The sCK level was within the reference range and the nerve conduction study revealed a mild sensory-motor polyneuropathy without myopathic changes at needle EMG. Cardiac examination by electrocardiography and ultrasound documented sinus tachycardia. A biopsy of vastus lateralis muscle showed slight fiber size variation, multiple cytoplasmatic bodies, many fibers harboring amorphous material, and several fibers with “rubbed-out” regions by NADH stain (Fig. 4 and S3). These abnormal fiber areas were strongly immunoreactive for antibodies to desmin, αB-crystallin, and myotilin. The patient died suddenly at the age of 55 years.

His sister (II-3) was examined at the age of 40 years because of difficulty in walking and raising from a chair probably started in young adulthood. On neurological examination, she had a waddling and steppage gait with mild trunk anteroflexion and scoliosis. She had a moderate weakness of hip flexors and severe weakness of foot extensors associated to proximal lower limb muscle wasting. At the upper limbs, a mild proximal weakness, especially of elbow extensors, and left scapular winging were observed, without significant muscle atrophy. No sensory, cerebellar, or cranial nerves involvement was present. Triceps and ankle deep tendon reflexes were absent. The patient noticed shortness of breath approximately from the age of 48 years when she started assisted ventilator at night. At the age of 54 years, she presented recurrent bouts of drug-refractory supraventricular tachycardia requiring ablation. Clinical examination at the age of 57 years showed severe weakness in almost all the muscles at four limbs. The sCK level was 2-fold above control value and EMG recorded a diffuse myopathic pattern. A needle biopsy of vastus lateralis muscle performed at the age of 41 years showed dystrophic features with marked fiber size variation, central nuclei, fiber splittings, few necrotic fibers, and perimysial and endomysial fibrosis. In addition, many fibers with “rubbed-out” areas were seen in NADH-stained sections. Family history was relevant for gait difficulties in their paternal aunt.

### Genetic investigations

**Fig. 5:**
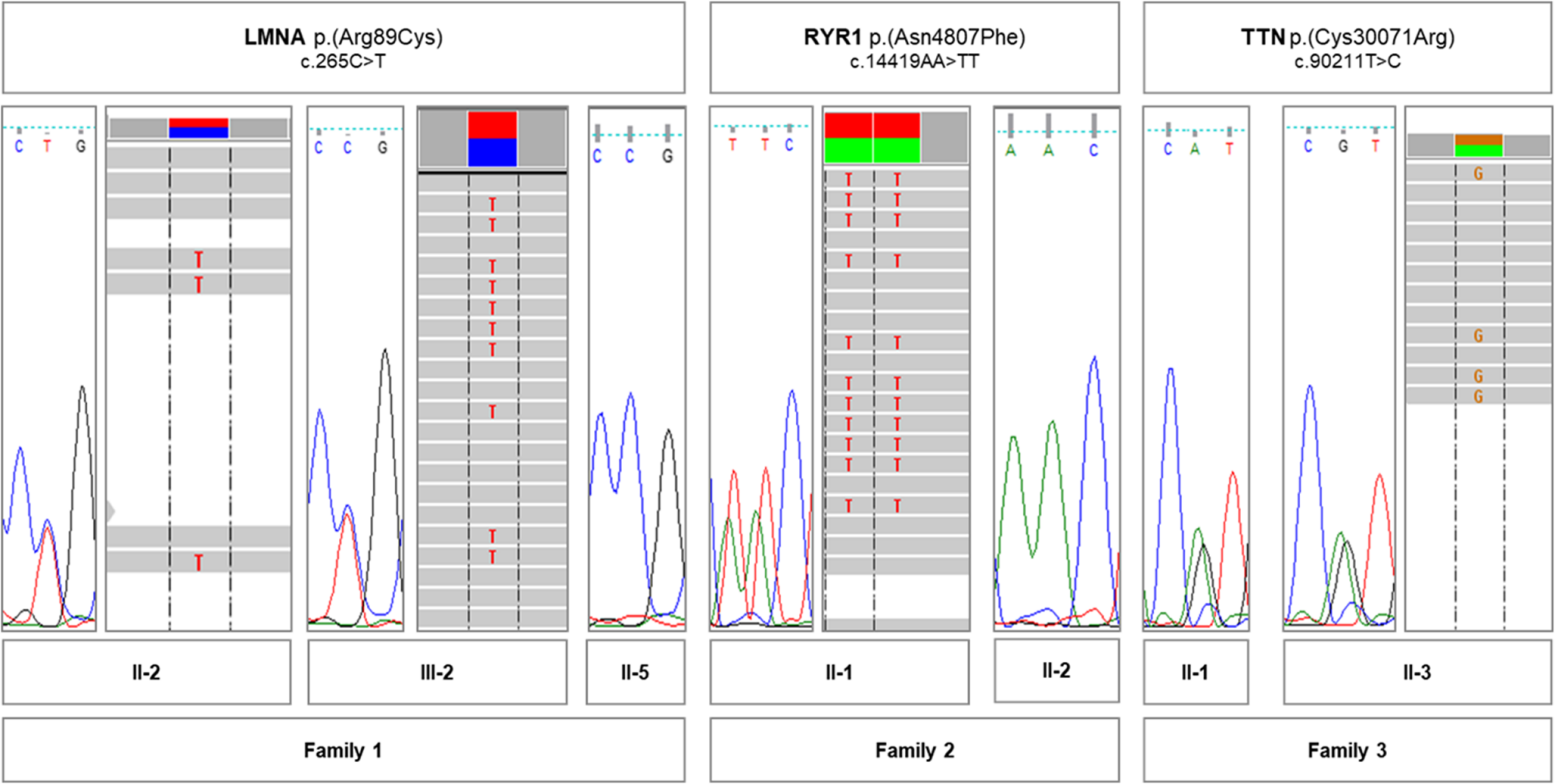
Candidate causative variants detected in three families. The Sanger sequencing peaks and Integrative Genomics Viewer screenshots are provided

The whole-exome data analysis identified candidate causative variants in all three families (Fig. 5 and Table 1) as well as several variants of unknown significance (VUS) (Table S1 and S2). Candidate causative variants in *LMNA* (Family 1), *RYR1* (Family 2), and *TTN* (Family 3) genes have null frequencies in gnomAD database as well as in our internal database. The p.(Arg89Cys) variant in *LMNA* gene is classified as “likely pathogenic” by the Varsome database according to the ACMG guidelines [19], together with the neighboring chr1:156084975-G>T (p.Arg89Leu) variant, which is also classified as pathogenic in the ClinVar database. Both *LMNA* variants have been described before in individuals with cardiomyopathy [20–22]. The RYR1 p.(Asn4807Phe) variant is caused by a dinucleotide substitution. Varsome lists several neighboring variants classified as pathogenic in ClinVar, including an adjacent p.(Phe4808Asn) variant (also caused by a dinucleotide substitution: chr19:39070679-TT>AA). Additionally, chr19:39070676-A>T, p.(Asn4807Tyr), and chr19-39070677:A>G, p.(Asn4807Ser) variants, located in the position of our dinucleotide variant and listed in dbSNP are both predicted to be damaging and are very rare in gnomAD Exomes. The TTN p.(Cys30071Arg) variant (NM_001256850.1), also described as p.(Cys31712Arg) (NM_001267550.2), has been previously shown to be damaging and causative [23, 24].

**Table 1:** Candidate causative variants detected in three families

Family proband	Variant (transcript) genomic pos. hg19	ACMG classification* (identified criteria)
Family 1	LMNA p.(Arg89Cys)/c.265C>T (NM_170707.4) chr1:156084974-C>T	Likely pathogenic (PM1, PM2, PM5, PP2, PP3)
II-2 (A)	+	
III-2 (A)	+	
II-5 (DP)	–	
Family 2	RYR1 p.(Asn4807Phe)/c.14419AA>TT (NM_000540.2) chr19:039070676-AA>TT	Uncertain significance (PM2, PP2, PP3)
II-1 (A)	+	
II-2 (U)	–	
Family 3	TTN p.(Cys30071Arg)/c.90211T>C (NM_001256850.1) chr2:179410829-A>G	Likely pathogenic (PM2, PP3, PP5, BP1)
II-3 (A)	+	
II-1 (A)	+	

*A*, affected; *U*, unaffected; *DP*, patient affected by myopathy with divergent phenotype

*Verdict and identified ACMG criteria are based on the Varsome database; *PS/PM/PP*, pathogenic strong/moderate/supporting; *BS/BP*, benign strong, supporting

### Immunohistochemistry

Immunofluorescence staining for lamin A/C revealed normal labeling of the nuclear envelope in proband of Family 1 as well as in controls (Fig. S4).

## Discussion

This study provides evidence that (1) p.(Arg89Cys) *LMNA* mutation causes a myopathy featuring PAM pathology, (2) *RYR1* mutations may be an additional cause of autosomal dominant PAM with an unusual phenotype of tibial myopathy, and (3) the p.(Cys30071Arg) mutation in TTN gene responsible for hereditary myopathy with early respiratory features (HMERF) is associated with cardiac involvement.

Lamins are intermediate filament proteins that constitute the nuclear lamina, a structure that underlies and provides mechanical support to inner nuclear membrane [25, 26]. Beside the structural role, nuclear lamins are involved in chromatin organization and transcription regulation as well as in physical connection between the nucleus and the cytoskeleton [25, 26]. *LMNA* encodes for lamin A and lamin C that results from alternative splicing of exon 10; lamins A and C are expressed in most differentiated cells [25, 26]. Despite mutations in *LMNA* have been associated with a heterogeneous group of diseases, known as the “laminopathies,” four main clinical phenotypes affecting skeletal and cardiac muscle have been reported including limb-girdle muscular dystrophy type 1B (LGMD1B), the Emery-Dreifuss muscular dystrophy (EDMD), congenital muscular dystrophy (MDCL), and dilated cardiomyopathy [25, 26]. Proband of Family 1 and her daughter harbored the same p.(Arg89Cys) mutation in *LMNA*. The mutation was previously reported in a patient with EDMD who had a different clinical phenotype and without pathological description [22]. Both members of our family clinically presented with arrhythmogenic cardiomyopathy and developed a proximal limb muscle weakness only in adulthood while in EDMD patient clinical disease onset was in early childhood with selective muscle involvement of the lower limbs and elbows contractures, cardiac conduction defects occurred at a later age. There was also no evidence of joint contractures in the mother and her daughter. MFM pathology has been reported in patients with *LMNA* myopathy but the occurrence is extremely rare and never associated with the described mutation [27, 28]. Interestingly, the brother of the proband who had a late-onset limb-girdle clinical phenotype was found to not carry the *LMNA* variant suggesting a mutation in a different gene or a sporadic or acquired myopathy. In addition, the patient did not develop an early arrhythmia unlike the proband and her daughter.

The *RYR1* gene encodes the skeletal muscle ryanodine receptor, a calcium channel that resides in the junctional sarcoplasmic reticulum (SR) membrane and, by interacting with the dihydropyridine receptor located on the T-tubules, releases calcium from the SR to the sarcoplasm in response to an action potential, triggering muscle contraction [29]. Autosomal dominant mutations in *RYR1* have been classically reported in patients with susceptibility to malignant hyperthermia (MH) and congenital central core disease (CCD) [30, 31]. Other RYR1-related phenotypes with both autosomal dominant and recessive inheritance pattern include multiminicore disease (MnD), centronuclear myopathy (CNM), congenital fiber type disproportion (CFTD), and King-Denborough syndrome (KDS) [30, 31]. Proband of family 2 was found to carry heterozygous missense mutation p.(Asn4807Phe) in *RYR1*, a variation which maps to the C-terminal MH3 domain of the protein. Several lines of evidence suggest that the *RYR1* variant identified in our patient is disease-causing; the variant was previously reported in a patient with CCD [32], it occurred at highly conserved positions, and it is predicted to have a damaging effect. Unfortunately, segregation analysis could not be performed because the proband’s father was dead and his old mother lived in a different region of the country. The onset and distribution of muscle weakness in our patient involving only distal lower limbs in the fourth decade of life is quite different from that observed in individual patients carrying the same *RYR1* variant [32] or the adjacent p.(Phe4808Asn) mutation [33–35] in which muscle weakness was proximal starting during early childhood [32, 34, 35] or there was only a susceptibility to MH without clinical myopathy [33]. In addition, he had a relevant cardiac history with atrial fibrillation which required ablation. Despite *RYR1* expression in cardiac muscle is limited, recently rare patients with cardiac involvement have been described [30].

Finally, we studied an Italian family and found the previously reported p.(Cys30071Arg) mutation in the *TTN* gene [23, 24]. *TTN* encodes the giant protein titin which spans through half of the sarcomere, with N- and C-terminal regions located at the Z-disk and M-line, respectively, and which is involved in thick filaments assembling and stabilization [36]. Titin sequence is characterized by fibronectin type III (FN3) and immunoglobulin-like modules that are repeated and organized in complex sequence arrangements [36]. The missense p.(Cys30071Arg) mutation occurs in exon 343 encoding for the FN3 domain 119 of the titin A-band, a region of the protein that establishes strong interactions with the thick filaments providing a molecular template for their assembling and represents the most common mutation in patients with HMERF, reported in more than 20 families [23, 24, 37–41]. The clinical manifestations and the histological features described in our family are consistent with HMERF phenotype, including autosomal dominant inheritance, adult onset, weakness in proximal, distal and respiratory muscles, and MFM pathology [23, 24, 37–41]. The significant involvement of cardiac muscle in our patients represents a new aspect of the disease. The proband died suddenly and cardiac investigations documented severe sinus tachycardia; furthermore, his sister developed heart conduction defects including several episodes of supraventricular paroxysmal tachycardia. HMERF does not appear to be associated with major cardiac involvement [42] but our observations suggest that cardiac surveillance should be recommended in patients carrying this mutation.

Additionally, we identified multiple VUS in other genes linked or not with myopathy besides the potentially causative mutations (Table S1). Novel variants in *DCTN1*, *MYH14*, and *RBM20* were identified in Family 1 with cardiac and limb-girdle muscle involvement and in *TRIM63*, *ACTC1*, and *TTN* in the proband of family 2 with a distal lower limb phenotype and atrial fibrillation. Clinical significance of these additional variants remains speculative and needs to be elucidated; they might contribute to the variability of the phenotype due to a synergistic or an antagonistic effect [43]. Alternatively, it has been suggested that the cumulative burden of variants might affect the functioning of target tissue modulating the penetrance and/or the expression of clinical phenotype [5].

In summary, whole-exome analysis in our PAM patients identified novel variants in known disease genes, a novel candidate disease gene, and documented phenotypic expansion for known causative genes.

## Supplementary Information

Figure S1 **Light microscopy of the muscle biopsy from proband of Family 1** (A and D) H&E stain shows fiber size variation, slight connective tissue proliferation, increased adipose tissue and two single vacuoles (asterisks). In two muscle fibers amorphous material stains dark blue or pink on GT (B and E, arrows). A few muscle fibers with focal and irregular decrease activity of NADH; no cores are observed (C). Images were obtained with obj × 20 (A-C) or obj × 40 (D-F). **Fig. S2. Light microscopy of the muscle biopsy from proband of Family 2** (A and B) H&E stain shows marked fiber size variation with atrophic and hypertrophic fibers, increase of endomysial connective tissue and two ring fibers. In two muscle fibers isolated amorphous material stains eosinophilic (arrows). A few muscle fibers with uneven, patchy loss of reactivity on NADH staining; no typical core lesions are present (C and D). Images were obtained with obj × 20. **Fig. S3. Light microscopy of the muscle biopsy from proband of Family 3** (A-D) NADH stain shows rubbed-out fibers and core-like lesions in several muscle fibers. Images were obtained with obj × 20. **Fig. S4. Immunofluorescence staining for lamin A/C** Lamin A/C staining is detected along the nuclear envelope in muscle of patient 1 (A) and in control muscle (C). Hoechst 33258 (B, D) staining depicts cell nuclei. Images were obtained with obj × 20 (DOCX 8468 kb)

Table S1 (PDF 253 kb)

Table S2 List of all rare variants identified (XLSX 232 kb)
