# Supplementary material for: Whole-exome sequencing in patients with protein aggregate myopathies reveals causative mutations associated with novel atypical phenotypes
Source: Neurol Sci. 2020 Nov 10;42(7):2819–27. doi: 10.1007/s10072-020-04876-7 (PMC7654353; doi:10.1007/s10072-020-04876-7)
Supplement: Supplementary file 1 — Light microscopy of the muscle biopsy from proband of Family 1 (A and D) H&E stain shows fiber size variation, slight connective tissue proliferation, increased adipose tissue and two single vacuoles (asterisks). In two muscle fibers amorphous material stains dark blue or pink on GT (B and E, arrows). A few muscle fibers with focal and irregular decrease activity of NADH; no cores are observed (C). Images were obtained with obj × 20 (A-C) or obj × 40 (D-F). Fig. S2. Light microscopy of the muscle biopsy from proband of Family 2 (A and B) H&E stain shows marked fiber size variation with atrophic and hypertrophic fibers, increase of endomysial connective tissue and two ring fibers. In two muscle fibers isolated amorphous material stains eosinophilic (arrows). A few muscle fibers with uneven, patchy loss of reactivity on NADH staining; no typical core lesions are present (C and D). Images were obtained with obj × 20. Fig. S3. Light microscopy of the muscle biopsy from proband of Family 3 (A-D) NADH stain shows rubbed-out fibers and core-like lesions in several muscle fibers. Images were obtained with obj × 20. Fig. S4. Immunofluorescence staining for lamin A/C Lamin A/C staining is detected along the nuclear envelope in muscle of patient 1 (A) and in control muscle (C). Hoechst 33258 (B, D) staining depicts cell nuclei. Images were obtained with obj × 20 (DOCX 8468 kb) [file 10072_2020_4876_MOESM1_ESM.docx]

**SUPPORTING INFORMATION**

**Table S1** Selected variants of unknown significance (VUS)

| **Family** Proband | **Variant 1**  (transcript)  genomic pos. hg19 | **Variant 2**  (transcript)  genomic pos. hg19 | **Variant 3**  (transcript)  genomic pos. hg19 |
| --- | --- | --- | --- |
| **Family 1** | **RBM20 p.(Val535Ile)**  c.1603G>A  (NM_001134363.2)  chr10:112557341-G>A | **DCTN1 p.(Ile196Val)**  c.586A>G (NM_004082.4)  chr2:074598723-T>C | **MYH14 p.(Arg1858Cys)**  c.5572C>T  (NM_001145809.2)  chr19:050805020-C>T |
| II-2 (A) | + | + | + |
| III-3 (A) | - | + | + |
| II-5 (DP) | - | NT | - |
| **ACMG Classification***  (identified criteria) | **Likely Benign**  (PP3, PP5, BS2, BP1) | **Benign**  (BS1, BS2, BP1, BP4, BP6) | **Likely Benign**  (PP3, BS2, BP1) |
| **Family 2** | **TRIM63 p.(Met14SerfsTer38)** c.35_38dupATCC  (NM_032588.3)  chr1:026393947-G>GGGAT | **TTN p.(Ser30125Phe)**  c.90374C>T  (NM_001256850.1) chr2:179410666-G>A | **ACTC1 p.(Ile371Thr)**  c.1112T>C (NM_005159.4) chr15:035082635-A>G |
| II-1 (A) | + | + | + |
| II-2 (U) | - | + | - |
| **ACMG Classification***  (identified criteria) | **Uncertain Significance**  (PM2) | **Benign**  (PP3, BS1, BS2, BP1, BP6) | **Uncertain Significance**  (PM2, PP3) |

A, affected; U, unaffected; DP, patient affected by myopathy with divergent phenotype; NT, not tested *verdict and identified ACMG criteria are based on Varsome database; PS/PM/PP – pathogenic strong/moderate/supporting; BS/BP – benign strong, supporting.

**FIGURE S1**

**
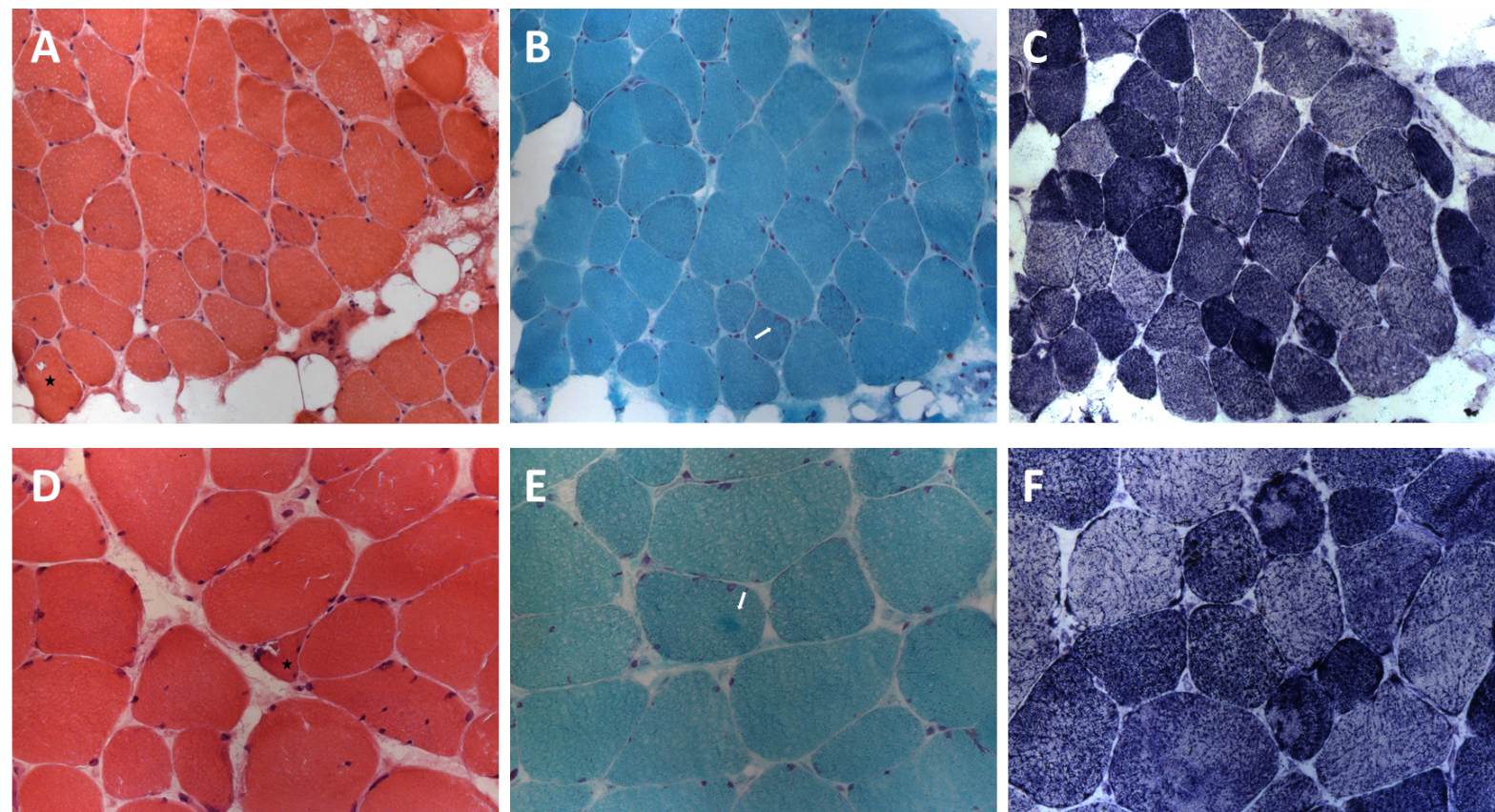
**

**FIGURE S2**

**
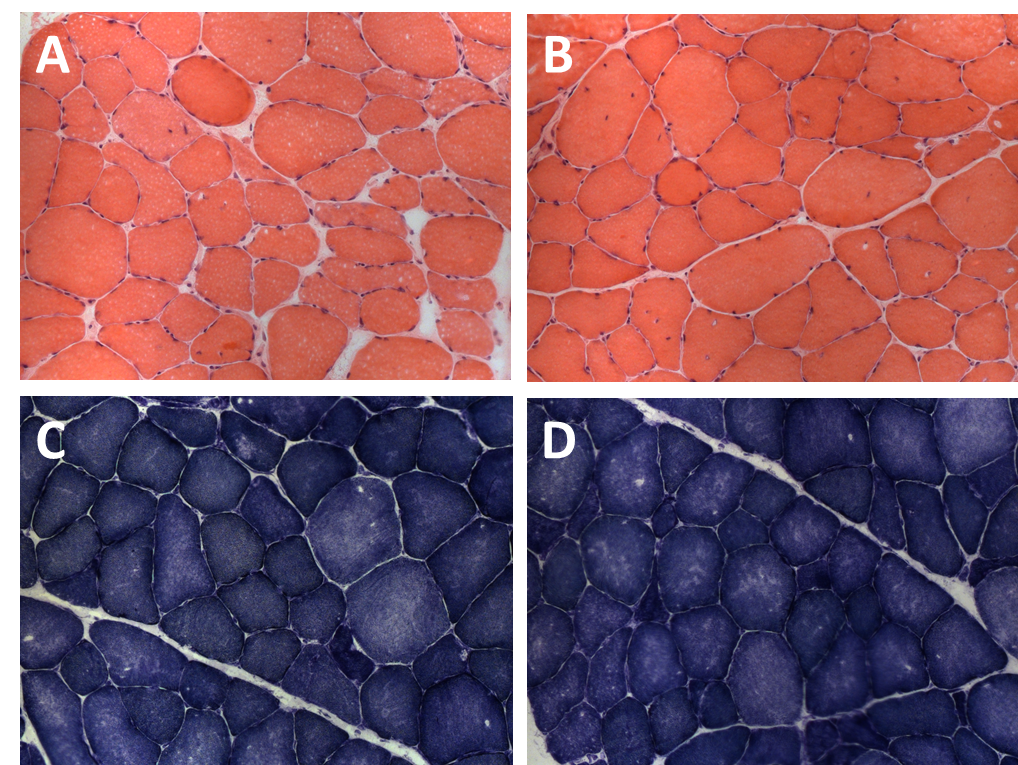
**

**FIGURE S3**

**
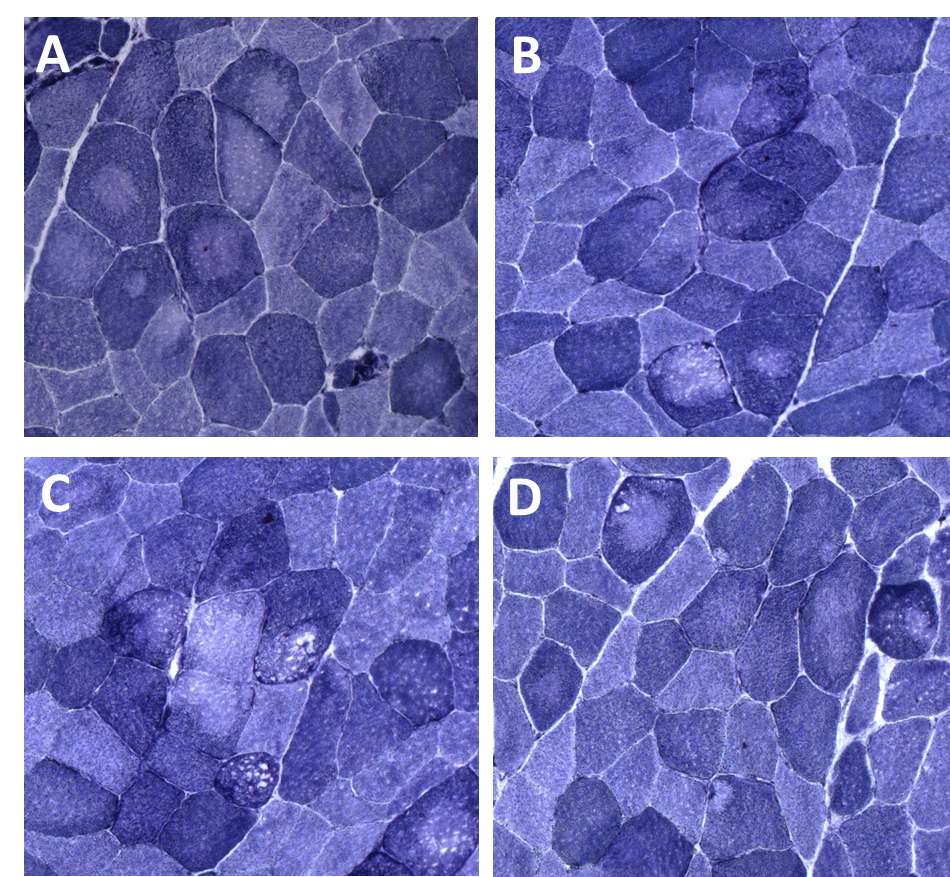
**

**FIGURE S4**

**
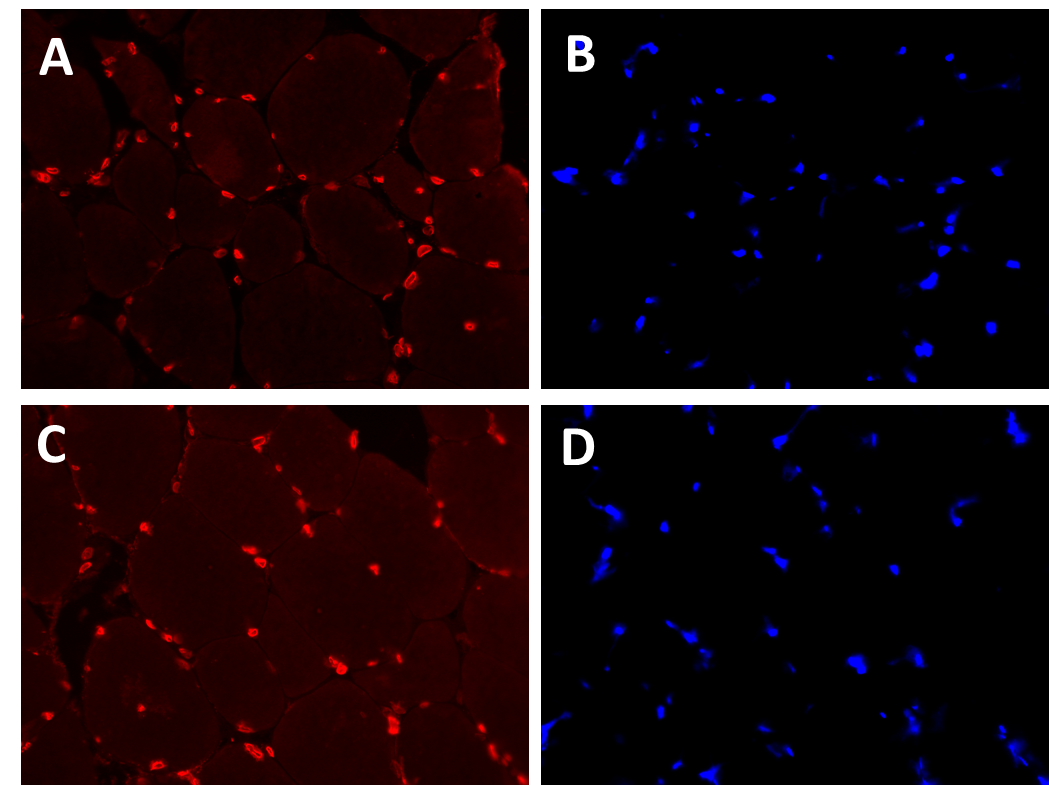
**
