# Supplementary material for: Whole-exome sequencing in patients with protein aggregate myopathies reveals causative mutations associated with novel atypical phenotypes
Source: Neurol Sci. 2020 Nov 10;42(7):2819–27. doi: 10.1007/s10072-020-04876-7 (PMC7654353; doi:10.1007/s10072-020-04876-7)
Supplement: Supplementary file 2 — (PDF 253 kb) [file 10072_2020_4876_MOESM2_ESM.pdf]

SUPPORTING INFORMATION

Table S2 Selected variants of unknown significance (VUS)

**Family**<br>Proband	**Variant 1**<br>(transcript)<br>genomic pos. hg19	**Variant 2**<br>(transcript)<br>genomic pos. hg19	**Variant 3**<br>(transcript)<br>genomic pos. hg19
**Family 1**	**RBM20 p.(Val535Ile)**<br>c.1603G>A<br>(NM_001134363.2)<br>chr10:112557341-G>A	**DCTN1 p.(Ile196Val)**<br>c.586A>G<br>(NM_004082.4)<br>chr2:074598723-T>C	**MYH14 p.(Arg1858Cys)**<br>c.5572C>T<br>(NM_001145809.2)<br>chr19:050805020-C>T
II-2 (A)	+	+	+
III-3 (A)	-	+	+
II-5 (DP)	-	NT	-
**ACMG Classification***<br>(identified criteria)	**Likely Benign**<br>(PP3, PP5, BS2, BP1)	**Benign**<br>(BS1, BS2, BP1, BP4, BP6)	**Likely Benign**<br>(PP3, BS2, BP1)
**Family 2**	**TRIM63 p.(Met14SerfsTer38)**<br>c.35_38dupATCC<br>(NM_032588.3)<br>chr1:026393947-G>GGGAT	**TTN p.(Ser30125Phe)**<br>c.90374C>T<br>(NM_001256850.1)<br>chr2:179410666-G>A	**ACTC1 p.(Ile371Thr)**<br>c.1112T>C<br>(NM_005159.4)<br>chr15:035082635-A>G
II-1 (A)	+	+	+
II-2 (U)	-	+	-
**ACMG Classification***<br>(identified criteria)	**Uncertain Significance**<br>(PM2)	**Benign**<br>(PP3, BS1, BS2, BP1, BP6)	**Uncertain Significance**<br>(PM2, PP3)

A, affected; U, unaffected; DP, patient affected by myopathy with divergent phenotype; NT, not tested
*verdict and identified ACMG criteria are based on Varsome database; PS/PM/PP – pathogenic strong/moderate/supporting; BS/BP – benign strong, supporting.
